# Intralesional Meglumine Antimoniate: Safe, Feasible and Effective Therapy for Cutaneous Leishmaniasis in Bolivia

**DOI:** 10.3390/tropicalmed7100286

**Published:** 2022-10-07

**Authors:** Ernesto Rojas Cabrera, Aleida Verduguez-Orellana, Igberto J. Tordoya-Titichoca, Ccoya Sejas, Rebeca Ledezma, Ingrid Álvarez, Jhonny Limachi-Choque, Nimer Ortuño-Gutiérrez, Marisol Córdova Rojas, Miguel Guzman-Rivero

**Affiliations:** 1 Centro Universitario de Medicina Tropical (CUMETROP), Universidad Mayor de San Simón, Cochabamba P.O. Box 3023, Bolivia; 2 Damián Foreign Foundation, Cochabamba P.O. Box 1256, Bolivia; 3 Leishmaniasis Regional Program, Secretary of Health, (SEDES, Cochabamba), Cochabamba P.O. Box 3000, Bolivia; 4 Epidemiology Unit, Military Hospital of the Corporación del Seguro Social Militar (COSSMIL), Cochabamba P.O. Box 1128, Bolivia; 5 Damien Foundation, Boulevard Léopold II, P.O. Box 263108 Brussels, Belgium

**Keywords:** cutaneous leishmaniasis, four-step strategy, intralesional treatment

## Abstract

The standard of care for cutaneous leishmaniasis includes the intramuscular/intravenous administration of pentavalent antimonials that are toxic and poorly tolerated. Primary health care usually lacks trained health staff for the diagnosis and treatment of leishmaniasis in Cochabamba Bolivia. Taking these aspects into account, a Bolivian consortium set out to explore the intralesional administration of meglumine antimoniate to treat cutaneous leishmaniasis during primary care under programmatic conditions. A four-step strategy consisting of clinical training for intralesional treatment and the promotion and periodic follow-up of health staff was carried out. The training process was applied in situ to personnel of nine primary health care centres. The intralesional treatment was applied five times every other day. Clinical follow-up after six-months of treatment showed a 77% healing proportion and 5% of therapeutic failure among 152 enrolled patients. The drug volume used in the intralesional procedure was on average 1.7 mL/ulcer treated. In conclusion, the strategy used was successful and effective, accomplishing a healing proportion similar to the long standardized treatment with a reduced time of administration, no severe side effects, and it is feasible to conduct by trained health staff. Our study supports the current PAHO/WHO recommendation for the intralesional administration of pentavalent antimonials for the treatment of cutaneous leishmaniasis.

## 1. Introduction

Cutaneous Leishmaniasis (CL) is the most frequent type of leishmaniasis surpassing visceral and mucocutaneous forms. It is estimated that the new cases of CL worldwide vary between 600,000 and one million [1]; 80% of the cases that have been diagnosed in Latin America come from Bolivia, Colombia, Peru, Nicaragua, and Brazil [2]. CL mainly causes skin ulcers in the exposed areas of the body, leaving scars and disabilities that cause stigmata [1]. Conventional cutaneous leishmaniasis treatments are conducted using the systemic application of pentavalent antimonials as the first drug option in different countries [3,4,5], including Bolivia. The recommended dosage is the daily application (intramuscular or intravenous) of 20 mg/kg/day for 20 days [4,6]. As a recommendation, the WHO/PAHO includes the use of intralesional application of this drug for cutaneous leishmaniasis treatment [7,8], based on evidence from studies of low certainty [8]. Currently, however, novel approaches to treatment are promising [9]. 

Different clinical trials evaluated the efficacy of the intralesional application of pentavalent antimonials and have shown variable healing proportions ranging from 56 to 93% [10,11,12,13,14,15,16]. On this matter, the Bolivian experience started with a proof-of-concept for single ulcers achieving a healing proportion of 70% [17]. Moreover, as a second option was available after the therapeutic failure of systemic treatments with meglumine antimoniate in 11 patients that had one or two ulcers covering an area equal or smaller than 900 mm^2^, where healing was accomplished in all cases [18]. Finally, in a quasi-experimental trial, three and six intralesional applications of meglumine antimoniate were compared, and healing was accomplished 30 days after the conclusion of treatment, independently of the number of applications, reaching 95% of clinical cures [19]. 

Primary health care centres are considered the initial contact of communities with the health care system, and they provide continuous medical assistance by resolving their health needs permanently [20,21], despite the limitations presented by these in developing countries [21,22,23,24]. A previous experience of the intralesional treatment of cutaneous leishmaniasis in a Brazilian primary health care centre, performed with 30 subjects, concluded that the procedure is simple, efficient, and safe, in spite of having a few local adverse effects [14]. Intralesional treatment also implies a reduction in management costs and systemic adverse effects [14], which are frequent in the conventional treatment of cutaneous leishmaniasis mainly in elder adults. As the evidence on this matter is scant, a consortium composed of Centro Universitario de Medicina Tropical (CUMETROP) of the Universidad Mayor de San Simón (Cochabamba, Bolivia), the Leishmaniasis Regional Program of the Secretary of Health of Cochabamba (Health Ministry, Bolivia), and Damian Foundation carried out this study in primary health care centres settled in different tropical areas to document the effectiveness, safety, and acceptance of the intralesional treatment of meglumine antimoniate under programmatic conditions. 

## 2. Materials and Methods

### 2.1. Study Population and Geographical Location 

The staff of nine primary health care centres (13 physicians and 8 nurses) participated, and they were recruited based on their annual report of leishmaniasis cases. Another aspect that was taken into account was their previous experience in collaborative work with CUMETROP in past experiences, and also an intense lobbying process was performed. The PHCCs were settled in two distinct geographical areas: 1. an endemic area for leishmaniasis, located in the Amazonian rainforest, and 2. in a non-endemic area located in inter-Andean valleys. Additionally, a pilot was installed in CUMETROP. Health facilities were selected under the following criteria: availability of health staff and the existence of the referral system and, in the case of an endemic area, the number of cutaneous leishmaniasis cases. 

Likewise, 152 cases with CL were recruited to receive the intralesional treatment with meglumine antimoniate. The number of recruited cases was taken into account based on the annual number of cases notified by the Cochabamba region to the Health Ministry National Leishmaniasis Program (300 cases per year) [25].

The inclusion criteria of subjects were as follows: no more than 2 ulcers; ulcer diameter no more than 900 mm^2^; no lymphangitis; no lesions on the face, ears, or neck; persons aged at least 12 [17,19]; and with prior laboratory confirmations of the presence of parasites at the lesion’s edges using smear and culture techniques described elsewhere [20].

### 2.2. Public Health Strategies of Intervention 

A four steps strategy was performed: first, clinical training of the health staff of the primary health care centres selected for the application of meglumine antimoniate intralesionally; second, lobbying for the incorporation of primary health care centres; third, intralesional treatment by primary health centres staff; fourth, periodic monitoring of trained staff through programmed visits to primary health care centres or by phone to resolve difficulties, retraining if necessary, and also the collection of information generated by PHCCs.

Training on the intralesional application of meglumine antimoniate was carried out for the health staff of PHCC in situ, which includes an oral presentation of the previous experiences in the matter and the demonstrative application procedure. In addition, an evaluation form of its clinical effectiveness was used, as defined by criteria described elsewhere [17,19]. Briefly, it was considered therapeutic failure when there was more than 50% growth of the original size or incomplete epithelialization 6 months after finishing treatment. Clinical effectiveness was considered relapse if it grows back more than 25% after it had decreased in size, or there is an appearance of new satellite lesions in the area surrounding the treated ulcer. Clinical effectiveness was considered clinical cure if there complete healing of all lesions was observed within six months after the completion of the treatment. 

### 2.3. Clinical Intervention 

The intralesional treatment comprised 5 applications conducted every other day during 9 days (days: 1; 3; 5; 7; 9). The decision to administer five applications is supported by results of previous essays [17,19] where three or six applications were fulfilled without showing substantial differences in the healing proportion.

The dose of the drug to be inoculated was calculated by multiplying the ulcer area by a mathematical factor of 0.008 [17].

Possible adverse effects were monitored during the treatment and also during clinical follow-up periods. Monitoring consisted in general clinical-assessment registration in the corresponding form as part of clinical records by the involved staff of PHCCs. 

The monitored adverse effects were as follows: myalgia, anorexia, asthenia, nausea, fever, headaches, burning sensation, and local transient pain [17,19]. Hepatic, renal, and cardiac functional tests were not conducted.

### 2.4. Ethics Permission 

All patients, or their tutors in the case of children, signed a written consent included in the study, which has the approval of the ethics committee of the Medicine Faculty, Universidad Mayor de San Simón, Bolivia. People who have been diagnosed with cutaneous leishmaniasis, who did not agree to enter the study and/or who did not meet the study inclusion criteria, received systemic treatments as per the current regulations of the Health Ministry of Bolivia. The participation of the involved PHCC had the authorization of this entity. 

### 2.5. Statistical Analysis 

The SPSS software was used. The ANOVA test was used for comparisons between ulcer areas before treatment and also to compare the clinical follow-up process by PHCC settled in different geographical places. Statistical significance was defined as *p* < 0.05.

## 3. Results

### 3.1. Promotion for the Voluntary Involvement of Primary Health Care Centres Staff

Two of the PHCCs that are located in the Amazonian rainforest are on the road, about 165 to 200 km away from Cochabamba. The other five are located in villages in the indigenous park. The distance from one site to the other is around 80 to 100 km. Two additional PHCCs were located in inter-Andean valleys at a distance of 10 to 15 km from Cochabamba (Table 1). 

An intensive lobbying meeting was held by the consortium with all trained individuals to obtain the maximum participation of PHCCs. Nevertheless, the incorporation of primary health care centre staff was gradual after the conclusion of the training period. During the first period, the staff of five PHCCs of the endemic area for CL joined: Four of them are located in the villages of the Indigenous Park in the Amazonian rainforest and “Chimore” (an urbanised town in the road of the Amazonian rainforest). They started the procedure of the intralesional application of meglumine antimoniate. In the second period, two additional centres in the same area were incorporated. Finally, in the third period, two more PHCCs located in urbanised towns in the inter-Andean valleys (non-endemic areas for CL) were incorporated, adding up to nine primary health care centres involved in the experience of the intralesional treatment of cutaneous leishmaniasis. CUMETROP, located in the urban area of Cochabamba, was included as a pilot centre for meglumine antimoniate intralesional applications since the first period (Table 1). 

### 3.2. Intralesional Antimony Treatment 

**Figure 1:**
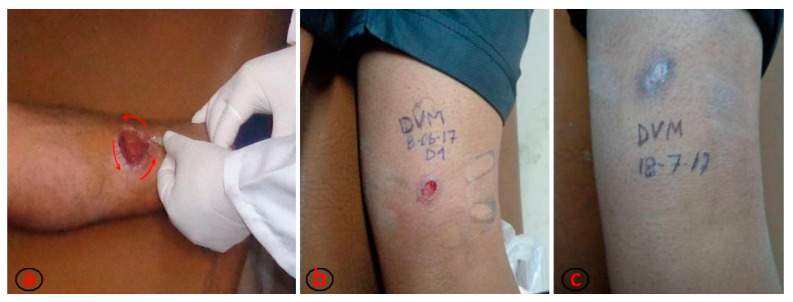
(**a**) Process of intralesional application of meglumine antimoniate on the ulcer borders until obtaining a whitened area around the ulcer. The total volume of drug to be applied for each ulcer is calculated by multiplying the ulcer area by the mathematical factor 0.008 [17]. (**b**) Ulcer before intralesional treatment. (**c**) Ulcer healed, corresponding to the first month after treatment conclusion. Pictures b and c are from the same patient. The photographs are property of Ernesto Rojas Cabrera (CUMETROP).

Each study subject recruited was administered five intralesional applications of meglumine antimoniate with an insulin syringe on the ulcer’s borders by trained staff in each primary health care centre (Figure 1). 

Of the total number of patients treated, 80% (122/152) were male, and the rest were female. Age was within a variable range, from 10 to 80 years, although this is a disagreement with the inclusion criteria (age mean (SD), 31 (13.6) years old). The anatomical areas most affected by the cutaneous leishmaniasis were the lower and upper limbs 63% (96/152) and 34% (52/152), respectively. The anterior thorax, the retro auricular region, and the gluteus (1% each) were affected less frequently. 

**Figure 2:**
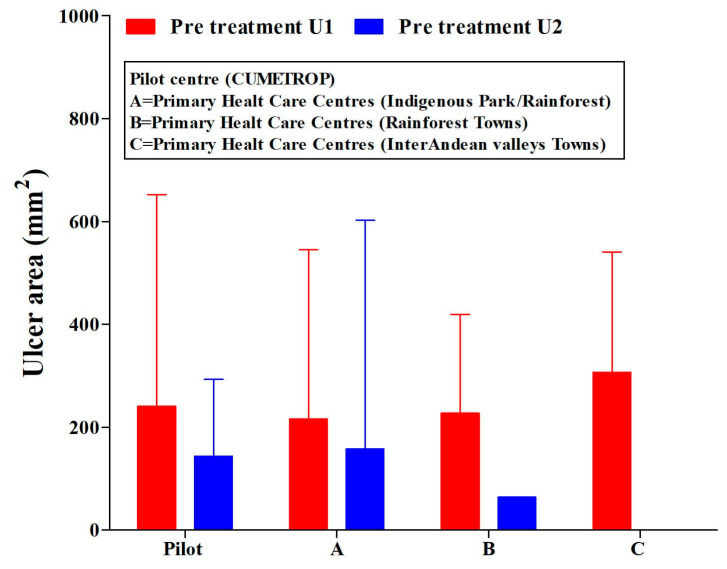
Ulcer areas (single or two ulcers) in cutaneous leishmaniasis patients. Pre-treatment with the intralesional application of meglumine antimoniate, regarding the settlement of primary health care centres and pilot centre. U1 = ulcer-1; U2 = ulcer-2. CUMETROP = Centro Universitario de Medicina Tropical. The data are presented as Mean (SD) ANOVA test (U1, *p* = 0.833; U2, *p* = 0.951).

The patients included in this study were treated in all PHCCs and the pilot centre (CUMETROP) previous to receiving intralesional application of meglumine antimoniate. They presented a great variability in their ulcer areas: independently, if there was a single ulcer or two ulcers (Figure 2) (ANOVA test, *p* > 0.05); Ulcer-1: range (2–1050) mm^2^; Ulcer-2: range (1–1600) mm^2^. 

Regarding adverse effects, only transient local pain was documented during the intralesional application in around 70% of patients in both groups (patients with single ulcer (81/117) and with two ulcers (25/35)). Moreover, possible adverse effects were monitored by the staff of PHCCs involved during the clinical follow-up period using a general clinical assessment, and the parameters indicated in the methodology were recorded. None of those adverse effects were found in that period.

The evaluation of the ulcer areas at the end of treatment period showed a few cases that in which the ulcer was healed (four cases for ulcer-1 and two cases for ulcer-2) and also those that increased ulcer areas by more than 900 mm^2^ (Table 2). 

Clinical follow-up after the completion of treatment has shown that around 50% of the ulcers in patients with a single or two ulcers healed completely (53/117 and 23/35 respectively) at the first post-treatment month, and the healing percentage increased at three and six months after the end of the treatment (Table 3). 

**Figure 3:**
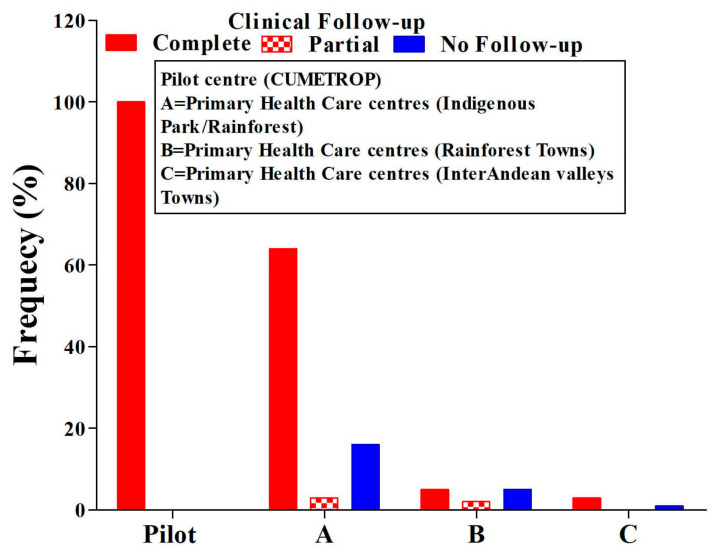
Comparison of ulcer conditions means clinical evaluation in the post-treatment follow-up period. ANOVA test, *p* = 0.01; CUMETROP = Centro Universitario de Medicina Tropical.

Healing, regardless of the presence of a single or two ulcers, was 77%. The lack of clinical follow-up in all PHCCs represents 17% (ANOVA test *p* = 0.01) (Figure 3). During the follow-up period, two cases of therapeutic failure were also identified in the first month after concluding treatment, and this progressed and reached eight cases ((8/152) 5%)) at six months post-treatment (Table 3). All cases identified as therapeutic failure were treated with systemic treatment, achieving complete healing at the end of the treatment. 

The volume of drug used was also evaluated, and this was on average 1.7 mL/ulcer treatment (1.83 mL (range 0.02 to 8.4 mL ulcer-1) and 1.49 mL (range 0.03 to 12.8 ulcer-2)). In all cases, the volume used is less than the standard volume used in the systemic treatment with the same drug.

## 4. Discussion

The recommendation of the Pan American Health Organisation (WHO Regional Office) regarding the treatment of cutaneous leishmaniasis in the Americas includes the intralesional application of antimonial drugs, which is to be carried out at the first or second level of care [8]. Likewise, primary health care centres are the closest to the people because they are in the communities, and even with common limitations in developing countries [21,22,23,24,25], the staff is close to the people and, therefore, have the trust of the communities [26]. Thus, both aspects argue in favour of the strategy used to motivate PHCC staff in the use of intralesional applications of meglumine antimoniate, which allowed the recruitment of 152 study subjects with cutaneous leishmaniasis who were able to solve their leishmaniasis with this procedure. These represent 17% of the cases reported per year (around 50/300 cases per year) by the regional leishmaniasis surveillance program to the Bolivian Health Ministry [27], and they are treated by the systemic procedure. The percentage of resolution by the intralesional procedure constitutes an important contribution if one takes into account that only nine PHCCs were involved in this experience. A remarkable aspect in this respect is the promotion of the timely resolution of cutaneous leishmaniasis in communities in a short period (9 days) compared with the twenty days required for systemic treatment [4,6]. Additionally, transient local pain was only found as an adverse effect during the intralesional application in around 70% of patients during treatment, and none during the clinical follow-up period, similarly to what we previously reported [17,19]. This situation probably responds to a minimal proportion of antimony present in the blood’s circulation after intralesional applications (less than 8 mL per ulcer/patient) for a short period (9 days), in contrast to systemic treatments in which serious adverse effects such as the electrocardiographic prolonged QTc interval and increased liver and pancreatic enzymes are commonly reported [28,29,30], mainly in elder people.

Another important result was the lobbying process, which was performed by our consortium with the PHCCs staff, achieving the progressive incorporation of nine centres that used the intralesional application of the drug. Perhaps the previous experience with the intralesional application of the drug in the area [17,19] contributed to the acceptance to participate in this experience, although there was a refusal from other centres despite the lobbying process. This situation likely reflects the natural mistrust of the staff towards a new procedure that is not included as a regular procedure in the therapeutic guidelines dictated by the Bolivian Health Ministry, even when there was express authorization from said ministry for the study, or perhaps there are other reasons such as the lack of motivation or incentives for these staffs by the health authorities, as found in another research study [31]. 

The strategy used also allowed the PHCC staff to comply with the selection criteria for patients with cutaneous leishmaniasis to receive intralesional treatments. There were offences in some respects by all participating centres, including the pilot centre. These offences are reflected in the inclusion of a 10-year-old patient, below the lower indicated age limit (12 years), who was cured with no severe adverse events reported during treatment. Moreover, offences also included the acceptance for the intralesional treatment of a patient with an ulcer in the retro auricular region and the acceptance of patients with ulcer areas greater than 900 mm^2^. These offences relative to the selection criteria represent 4% (6/152 cases), but in 96% (146/152 cases), the recommendations given during the training and follow-up process regarding the selection criteria of cutaneous leishmaniasis patients for the intralesional treatment were fulfilled and also reflect the acceptance of the training process and the intralesional procedure by the PHCC staff. The inclusion of patients with ulcer areas greater than 900 mm^2^ likely reflects a personal decision of physicians who took into account that the total volume of the drug used was less than 15 mL, which is considered the maximum volume that can be inoculated per day in the systemic treatment [32]. In addition, the size of the ulcers, most of which do not exceed 900 mm^2^, reflects the characteristics of cutaneous leishmaniasis ulcers in that region and supports the use of an intralesional application of the drug with smaller volumes, directly contributing to minimising possible adverse effects that are common in systemic treatments [28,29,30], mainly in elderly people, and it indirectly contributes to money saving with respect to the Bolivian Health Ministry, as it is responsible for providing treatments as part of the leishmaniasis control program because there is an effective reduction in the number of ampoules used in the treatment of cutaneous leishmaniasis (intralesional treatment: 360 ampoules used in 50 patients with a single ulcer (area = 900 mm^2^) vs. systemic treatment: 3000 ampoules used in the same 50 patients with a single ulcer (area = 900 mm^2^). This reduction should be seen not only for its ability to save money for the health ministry but also for its direct impact on patients’ income, as it reduces the number of daily commutes for patients receiving their treatments. Therefore, intralesional treatments should be considered important strategies for the treatment of cutaneous leishmaniasis as the first line of treatment in Bolivia due to savings in social costs and its impact on the budget [33]. 

The empowerment achieved by the staff of these nine PHCCs as a result of the four-step strategy used, which focused on intralesional treatment with meglumine antimoniate, is remarkable, since the staff, based on their own experience, assumed this procedure as a valid therapeutic procedure.

This study did not include a control group that would allow a more effective comparison of the achievements described here. Even so, the results obtained with the intralesional procedure should be considered as an important achievement, since a 77% healing proportion was achieved six months after the end of the treatment, a percentage that is within the healing proportion range obtained with the systemic treatment of cutaneous leishmaniasis with antimonial compounds [34,35].

Clinical follow-up after treatment is an important component of leishmaniasis control since it is possible to detect therapeutic failures and also identify the appearance of mucosal or mucocutaneous leishmaniasis in this period because *Leishmania braziliensis* is the most frequent species in Bolivia and is responsible for mucosal leishmaniasis [36,37,38]. In this manner, the training and periodic monitoring of the trained staff emphasised compliance with the clinical follow-up after the conclusion of the intralesional application of the drug to patients with cutaneous leishmaniasis. The results show, in this regard (Figure 3), that the compliance of the clinical follow-up failed in all PHCCs compared to the pilot centre (ANOVA test, *p* = 0.01). The complete clinical follow-up achieved globally was 77% (117/152); partial clinical follow up was 4% (6/152), and no clinical follow up represented 19% (29/152). The critical offences to the intervention strategy by the PHCC staff was the absence of clinical follow-ups in a significant percentage, reflecting the difficulty PHCC staff face in carrying out a prolonged period of clinical follow-up of patients with cutaneous leishmaniasis after having completed their treatments, even though they know the importance of this activity, as stated above. It was not possible to identify the reasons why the staff of the involved PHCC had difficulty in performing regular clinical follow-up after the conclusion of treatment for patients with cutaneous leishmaniasis; this, situation could be explained by the workload in primary health care centres, as indicated by other studies [39,40]. 

The clinical follow-up period, despite the difficulties mentioned above, allowed the progressive observation of cases of therapeutic failure. In the first month after finishing the treatment, we found that five cases in total for both ulcers and reached eight cases (5%) at 6-months post-treatment. It is important to highlight that therapeutic failure is also present in systemic treatments in different proportions, attributable to factors varied by the authors [35,41,42], and is close to the failure rate that we observed with intralesional administrations.

## 5. Conclusions

This is the first study in Bolivia that demonstrates that the intralesional administration of antimonial meglumine was clinically effective, since a 77% healing proportion was achieved six months post-treatment. It is also feasible, as demonstrated by this study, under real programmatic conditions of PHCCs, including its limitations. Moreover, intralesional treatments were safe because no severe adverse effects were reported, with pain being the mild adverse event reported in 70% of cases.

Our strategy to motivate the PHCC staff in the use of the intralesional application of meglumine antimoniate allowed the recruitment of 17% of cutaneous leishmaniasis patients of the notified cases per year, by the regional program of leishmaniasis surveillance to the Bolivian Health Ministry, and the resolution of leishmaniasis in the patients in a short period of time (9 days) compared with the twenty days required by systemic treatments; adverse effects of this type of treatment were avoided.

The strategy used allowed the promotion of the timely resolution of cutaneous leishmaniasis in communities by intralesional treatments, as well as the progressive incorporation of primary health care centres for the use of intralesional procedures as a valid therapeutic procedure. 

The size of ulcers that mostly do not exceed 900 mm^2^ support the use of the intralesional procedure, allowing the Bolivian Health Ministry to save money. 

Greater knowledge was achieved with respect to the importance of clinical follow-up after treatment because it allowed identifying cases of therapeutic failure, although there are difficulties in compliance.

## Figures and Tables

**Table 1:** Distribution of primary health care centres involved in the meglumine antimoniate intralesional application in intervention period.

First Period (2019)		Second Period (2020)		Third Period (2021)	
Primary Health Care Centre	Settlement Region	Primary Health Care Centre	Settlement Region	Primary Health Care Centre	Settlement Region
Chipiriri	Villages in the Indigenous Park /Amazonian rainforest	Santísima Trinidad	Villages in the Indigenous Park /Amazonian rainforest	El Abra	Urbanized town/ inter-Andean valleys
Ichoa					
Independencia					
Tacopaya					
Chimoré	Urbanized town/Amazonian rainforest	Entre Ríos	Urbanized town/Amazonian rainforest	Ironcollo	
Pilot Centre: CUMETROP (Cochabambacity; inter-Andean valleys)					

Amazonian rainforest: endemic area for leishmaniasis. Inter-Andean valleys: non-endemic area for leishmaniasis and receptor of patients from endemic areas.

**Table 2:** Patients with single or two ulcers and their conditions at the end of the intralesional treatment period, with meglumine antimoniate by the settlement of primary health care centres. Data presented as frequencies (%).

Ulcer Conditions at the End of the Intralesional Treatment		Primary Health Care Centres Settlement			
		Villages in the Amazonian Rainforest/Indigenous Park	Urbanised Town in the Amazonian Rainforest	Urbanised Town in the Inter-Andean Valleys	CUMETROP (Pilot Centre), Cochabamba City
Patient with single ulcer *n* = 117	Healing	2 (2)	1 (1)	-	1 (1)
	Ulcer area partial reduction	83 (71)	6 (5)	5 (4)	13 (11)
	Ulcer area enlargement	3 (3)	1 (1)	-	2 (1)
Patients with two ulcers *n* = 35	Healing	2 (6)	-	-	-
	Ulcer area partial reduction	26 (74)	1 (3)	-	5 (14)
	Ulcer area enlargement	1 (3)	-	-	-

Amazonian rainforest: endemic area for leishmaniasis. Inter-Andean valleys: non-endemic area for leishmaniasis and receptor of patients from endemic areas. Dash (-): no recorded ulcers.

**Table 3:** Patients with single or two ulcers and their conditions during clinical follow-up after treatment with intralesional meglumine antimoniate. Data presented as frequencies (%).

Ulcer Conditions		First Month Post-Tx.	Third Month Post-Tx.	Sixth Month Post-Tx.
Patient with a single ulcer *n* = 117	Healing	53 (45)	70 (60)	85 (73)
	Partial reduction	30 (26)	13 (11)	-
	Without changes	2 (2)	5 (4)	-
	Increasing	3 (3)	1 (1)	-
	Withdrawal	1(1)	-	-
	Therapeutic Failure	2 (2)	1 (1)	5 (4)
	No follow up	26 (22)	27 (23)	27 (23)
Patients with two ulcers *n* = 35	Healing	23 (66)	29 (83)	31 (89)
	Partial reduction	7 (20)	2 (6)	-
	Without changes	1(3)	-	-
	Increasing	-	-	-
	Withdrawal	-	-	-
	Therapeutic Failure	-	-	1 (3)
	No follow up	4 (11)	4 (11)	3 (9)

Post-Tx.: post treatment. Dash (-): No recorded ulcers.

## Data Availability

The data presented in this study are available on request from the corresponding author.
